# SARS-CoV-2 aerosol generation during respiratory equipment reprocessing

**DOI:** 10.1186/s13756-021-00955-2

**Published:** 2021-05-27

**Authors:** Camila Quartim de Moraes Bruna, Caroline Lopes Ciofi-Silva, Anderson Vicente de Paula, Lucy Santos Villas Boas, Noely Evangelista Ferreira, Tania R. Tozetto-Mendoza, Maria Cássia Mendes Correa, Kazuko Uchikawa Graziano

**Affiliations:** 1 Escola de Enfermagem da Universidade de São Paulo, Av Dr Enéas de Carvalho Aguiar, 419, Cerqueira Cesar, São Paulo, SP 05403-000 Brazil; 2 PETIRAS Research Group, São Paulo, Brazil; 3 Faculdade de Medicina da Universidade de São Paulo, Laboratorio de Investigação Médica em Virologia (LIM52), Instituto de Medicina Tropical de São Paulo, Av Dr Enéas de Carvalho Aguiar, 470, Cerqueira Cesar, São Paulo, SP 05403-000 Brazil

## Abstract

Aerosolization may occur during reprocessing of medical devices. With the current coronavirus disease 2019 pandemic, it is important to understand the necessity of using respirators in the cleaning area of the sterile processing department. To evaluate the presence of severe acute respiratory syndrome coronavirus (SARS-CoV-2) in the air of the sterile processing department during the reprocessing of contaminated medical devices. Air and surface samples were collected from the sterile processing department of two teaching tertiary hospitals during the reprocessing of respiratory equipment used in patients diagnosed with coronavirus disease 2019 and from intensive care units during treatment of these patients. SARS-CoV-2 was detected only in 1 air sample before the beginning of decontamination process. Viable severe acute respiratory syndrome coronavirus 2 RNA was not detected in any sample collected from around symptomatic patients or in sterile processing department samples. The cleaning of respiratory equipment does not cause aerosolization of SARS-CoV-2. We believe that the use of medical masks is sufficient while reprocessing medical devices during the coronavirus disease 2019 pandemic.

## Background

With the current coronavirus disease 2019 (COVID-19) pandemic, some of the discussions are focused on the use of personal protection equipment (PPE), especially masks and respirators. The general recommendation of using coveralls with foot covers [1] and respirators led to PPE shortage [2].

COVID-19 affects the primary respiratory tract, and although most patients have favorable progression, approximately 20% develop severe forms of the disease, requiring ventilatory assistance using non-invasive ventilation or orotracheal intubation [3, 4]. Thus, respiratory therapy equipment is considered semi-critical items [5]; therefore, when not disposable, these materials should be reprocessed. Reprocessing includes manual or mechanical cleaning and high-level disinfection or sterilization, which are activities that can disperse droplets and even produce aerosols in the sterile processing department (SPD) decontamination area [6].

The guidelines for SPD workers recommend the use of a fluid-resistant face mask and eye protection [7]. However, information on the use of special respirators, such as N95 and PFF2 masks, is lacking, and to the best of our knowledge, no special guideline has been established for these workers during the pandemic, perhaps because they are not involved in direct care.

As with other health-care workers, SPD personnel are concerned about working in a place where possible severe acute respiratory syndrome coronavirus 2 (SARS-CoV-2) droplets could be generated. Considering the shortage of respirators and the possible dispersal of the virus in the form of an aerosol (aerosolization) during the cleaning of SARS-CoV-2–contaminated material in the SPD, the aim of this study was to evaluate the presence of SARS-CoV-2 aerosols in the air of SPDs during the reprocessing of contaminated respiratory therapy equipment.

## Methods

### Setting

This cross-sectional study was performed in 2 teaching tertiary hospitals from July to August 2020.

### Air sample collection

Air samples were collected in COVID intensive care units (ICUs) and SPD from both hospitals. For the ICU samples, the air was collected from patients with invasive mechanical ventilation and non-invasive oxygen therapy. These patients were positive RT-PCR results for SARS-CoV-2 carried out between 2 and 5 days after onset of symptoms and collected up to 72 h before the sampling day.

In the SPD, the air was collected always in 3 different time points in the decontamination area: (1) before the beginning of the decontamination processes; (2) for the mechanical cleaning, during unpacking and assembling of the respiratory equipment in the washer racks; (3) for the manual cleaning, during unpacking, brushing, and rinsing of the respiratory equipment. The Table 1 shows the study design and number of samples in each setting. The time from retrieval of the respiratory equipment from the patient to air collection varied from 30 min to 2 h.

**Table 1:** Method design and sample characteristics

Sample characteristics	Number of samples
ICU	
Hospital A: near intubated patients	3
Hospital A: during changing of mechanical ventilation lines	1
Hospital B: near symptomatic patients with low-flow nasal cannula or without oxygen therapy devices	2
SPD	
Hospital A: before decontamination (negative control)	3
Hospital A: mechanical cleaning	5
Hospital B: before decontamination (negative control)	3
Hospital B: manual cleaning	5
Swabs	
Swabs from respiratory equipment lines of Hospital B	3
Total of samples	25

Values are presented as numbers

*ICU* intensive care unit, *SPD* sterile processing department

None of the ICU or decontamination areas of the SPD were negative-pressure rooms or had an air conditioning system. The SPDs from hospitals A and B measure 19.84 and 42 m^3^, respectively. The windows and doors were kept closed during all experiments.

### Air sampler

The Coriolis^®^µ air sampler (Bertin Technologies, France), which was set at 300 L/min for 10 min, was placed 20 inches (50 cm) from the respiratory therapy equipment being manually cleaned or during the assembly of the equipment in the washer racks or 20 inches (50 cm) from the patients’ face. A sterile cone coupled to the air sampler was prefilled with 15 mL of 0.9% saline. This volume was then transferred to a centrifugal filter unit (Amicon-Ultra15, 30 kDa, Merck-Millipore, Germany) and centrifuged for 10 min at 5000 rpm at 4 °C.

### Swab collection

Swab samples were obtained from the interior of the silicone respiratory equipment lines used by patients recently diagnosed with COVID-19. Sampling was performed using sterile flocked nylon swabs (FloqSwabs; Copa, Italy). Three swabs were rotated and rubbed over each proximate end of the respiratory lines in a zigzag pattern. The swab tips were then placed in a tube with 10 mL of 0.9% saline.

### SARS-CoV-2 detection

Nucleic acid extraction was performed using a QIAmp viral RNA mini kit (QIAGEN, Germany). Samples were then subjected to RT-PCR (RealStar^®^ SARS-CoV-2 RT-PCR Kit 1.0; Altona Diagnostics, Germany) followed by DNA amplification (Roche LightCycler^®^ 96 System; Roche Diagnostics, Switzerland). Aliquots of the samples were inoculated in Vero cells (ATCC^®^ CCL-81™; ATCC, USA) in Dulbecco’s minimal essential medium supplemented with 10% heat-inactivated fetal bovine serum and antibiotics/antimycotics and incubated in a 37 °C incubator in an atmosphere of 5% CO_2_. Cultures were maintained for at least 2 weeks and observed daily for evidence of cytopathic effects (CPEs). At least 2 subcultures were performed on each sample. CPEs were detected using an inverted microscope (Nikon, Japan), and the presence of virus in supernatants from cultures showing CPEs was determined by specific RT-PCR, as described above. RT-PCR analysis was performed using RNA extracted from culture supernatants obtained after 2 passages after the initial inoculation.

### Ethical considerations

This study was approved by the Ethics and Research Committee (CAAE 35133120.1.0000.5392).

## Results

SARS-CoV-2 RNA was detected only in 1 air sample, in hospital B, before the beginning of decontamination processes, showing a Ct value of 36.88, but the cell cultures were negative. The swab collection also showed no detection of the virus.

## Discussion

We obtained air samples in places where we supposed SARS-CoV-2–contaminated aerosol or droplets would have been generated (clinical areas with symptomatic patients and during the cleaning of respiratory equipment used by these patients). However, viral RNA was identified in only 1 air sample collected in the SPD, even when no decontamination procedure was performed in the area.

Some authors have isolated the virus from aerosol and other surfaces in experimental controlled conditions [8, 9], whereas others have detected SARS-CoV-2 RNA in air samples during endotracheal intubation [10], in areas where aerosol-generating procedures (AGPs) were performed, and in other public areas without known COVID-19 patients [11]. Nevertheless, until now, only 1 study has identified viable SARS-CoV-2 in air samples collected in a room occupied by patients diagnosed with COVID-19 but in the absence of an AGP [12, 13]. Air contamination with SARS-CoV-2 and the risk of airborne transmission still need further researches, as the possible contamination and viability of the in respiratory equipment lines.

During cleaning, the surfactants in detergents can dissolve the lipid bilayer membrane of SARS-CoV-2, providing a virucidal action [14]. This may explain the absence of aerosolization during manual cleaning in this study. Additionally, as SARS-CoV-2 RNA was not detected in respiratory equipment lines, the virus could probably become non-viable on the equipment after being removed from the patient. In another study, viral RNA was identified in cooling water from ventilator circuits, but no culture was performed [10].

Proper ventilation and air control may reduce viable virus from air samples, which can lead to false-negative air samples that do not represent the real AGP situation [15]. The positive sample of this study can be explained by airflows or faults in ventilation systems. It is worth mentioning that this sample was weakly positive (Ct 36.88) [15].

We acknowledge that the air sampling method used in this study has limitations. The viral particles could be destroyed by the Coriolis^®^ µ equipment during air aspiration and centrifugation. However, other air sampling methods have been previously used, and it has not been possible to capture viable SARS-CoV-2 particles either [15]. Other limitations are the low number of samples and the lack of measurement of aerosol size.

## Conclusion

We were unable to find SARS-CoV-2 aerosols in the air of SPDs during the reprocessing of contaminated respiratory therapy equipment. Based on these results, we conclude that the respiratory equipment might not have viable SARS-CoV-2 after use, hence the routine tasks in the SPD cannot be considered an AGP.

## Data Availability

Data analysed during the current study are available from the corresponding author (CQMB caquartim@yahoo.com.br) on reasonable request, as long as this meets local ethics and research governance criteria.

## Abbreviations

SARS-CoV-2: Severe acute respiratory syndrome coronavirus 2
COVID-19: Coronavirus disease 2019
PPE: Personal protection equipment
SPD: Sterile processing department
PFF2: Particulate filtering facepiece
ICU: Intensive care units
RT-PCR: Reverse transcription–polymerase chain reaction
DNA: Deoxyribonucleic acid
CPE: Cytopathic effects
RNA: Ribonucleic acid
AGP: Aerosol-generating procedures
Ct: Threshold cycle
